# Supplementary material for: Impact of CYP2C9 and VKORC1 Polymorphisms on Warfarin Sensitivity and Responsiveness in Jordanian Cardiovascular Patients during the Initiation Therapy
Source: Genes (Basel). 2018 Nov 27;9(12):578. doi: 10.3390/genes9120578 (PMC6316567; doi:10.3390/genes9120578)
Supplement: Supplementary file 1 [file genes-09-00578-s001.pdf]

Table S1: Single Nuvleotide Polimorphisms (SNPs) ID, their position and genotyping data based on whole cohort ($N = 220$).

Gene	SNP ID	Chr Position[a]	SNP	SNP Type	Discrepancy rate[b]	Call rate[c]
VKORC1	rs104894539	16:31094645	C/A	Missense variant	0.06%	96%
	rs104894540	16:31094596	A/G	Missense variant	0.04%	96%
	rs104894541	16:31094558	T/C	Missense variant	0.07%	96%
	rs104894542	16:31091243	A/C	Missense variant	0.05%	96%
	rs61742245	16:31094624	C/A	Missenses variant	0.03%	97%
	rs10871454	16:31036758	C/T	Exonic variant	0.12%	97%
	rs8050894	16:31093188	G/C	Intronic variant	0.03%	97%
	rs9934438	16:31093557	G/A	Intronicvariant	0.12%	96%
	rs17708472	16:31094032	C/T	Exonic variant	0.00%	97%
CYP2C9	rs1799853	10:94942290	C/T	Missense variant	0.15%	96%
	rs28371685	10:94981224	C/T	Missense variant	0.07%	97%
	rs28371686	10:94981301	C/G	Missense variant	0.05%	97%
	rs4086116	10:94947445	C/T	Intronic variant	0.04%	96%
	rs72558191	10:94947919	T/G	Missense variant	0.07%	97%
	rs9332131	10:94949282	Del	Frameshift variant	0.05%	96%
	rs9332239	10:94989020	C/T	Missense variant	0.03%	97%
	rs1057910	10:94981296	A/C	Missense variant	0.03%	97%

[a.] Chromosome positions are based on NCBI Human Genome Assembly Build.
[b.] Ratio of the number of discordant genotypes to the number of duplicates.
[c.] Ratio of the number of valid genotypes to the number of subjects genotyped ($n = 220$) at each locus

Table S2: List of SNPs, their minor allele frequencies, and HWE *p* values for genotypic distribution at each locus based on 220 patients

Gene	SNP ID	MA[a]	MAF[b]	HWE[c] *p value*
VKORC1	rs104894539	A	n/a*	n/a*
	rs104894540	G	n/a*	n/a*
	rs104894541	C	n/a*	n/a*
	rs104894542	C	n/a*	n/a*
	rs61742245	A	n/a*	n/a*
	rs10871454	C	0.48	0.568
	rs8050894	C	0.47	0.525
	rs9934438	G	0.49	0.487
	rs17708472	A	0.13	0.730
CYP2C9	rs1799853	T	0.12	0.531
	rs28371685	T	n/a*	n/a*
	rs28371686	G	n/a*	n/a*
	rs4086116	T	0.23	0.550
	rs72558191	G	n/a*	n/a*
	rs9332131	Del	n/a*	n/a*
	rs9332239	T	n/a*	n/a*
	rs1057910	C	0.1	0.119

[a.] MA: Minor allele.

[b.] MAF: Minor allele frequency.

[c.] HWE: Hardy-Weinberg equilibrium.

*n/a: not applicable (nonpolymorphic)

Table S3: The frequency of the allele and genotype for all polymorphisms in cardiovascular patients treated with warfarin.

Gene	SNP ID	Allele/Genotype	% (*n*)
VKORC1	rs104894539	C	100% (*n* = 422)
		A	0%
		CC	100% (*n* = 211)
		CA	0%
		AA	0%
	rs104894540	A	100% (*n* = 422)
		G	0%
		AA	100% (*n* = 211)
		AG	0%
		GG	0%
	rs104894541	T	100% (*n* = 424)
		C	0%
		TT	100% (*n* = 212)
		TC	0%
		CC	0%
	rs104894542	A	100% (*n* = 424)
		C	0%
		AA	100% (*n* = 212)
		AC	0%
		CC	0%
	rs61742245	C	99% (*n* = 420)
		A	1% (*n* = 4)
		CC	98% (*n* = 208)
		CA	2% (*n* = 4)
		AA	0%
	rs10871454	C	48% (*n* = 204)
		T	52% (*n* = 220)
		CC	22% (*n* = 47)
		CT	52% (*n* = 110)
		TT	26% (*n* = 55)

Table S3 (continued)

Gene	SNP ID	Allele/Genotype	% (*n*)
	rs8050894	C	46% (*n* = 196)
		G	54% (*n* = 228)
		CC	20% (*n* = 43)
		GC	52% (*n* = 110)
		GG	28% (*n* = 59)
	rs9934438	G	49% (*n* = 207)
		A	51% (*n* = 217)
		GG	23% (*n* = 48)
		AG	52% (*n* = 111)
		AA	25% (*n* = 53
	rs17708472	G	87% (*n* = 369)
		A	13% (*n* = 55)
		GG	75% (*n* = 160)
		GA	23% (*n* = 49)
		AA	2% (*n* = 3)
CYP2C9	rs1799853	C	88% (*n* = 374)
		T	12% (*n* = 50)
		CC	77% (*n* = 164)
		CT	22% (*n* = 46)
		TT	1% (*n* = 2)
	rs28371685	C	99.7% (*n* = 423)
		T	0.3% (*n* = 1)
		CC	99.5% (*n* = 211)
		CT	0.5% (*n* = 1)
		TT	0%
	rs28371686	C	100% (*n* = 424)
		G	0%
		CC	100% (*n* = 212)
		CG	0%
		GG	0%

Table S3 (continued)

Gene	SNP ID	Allele/Genotype	% (*n*)
	rs4086116	C	77% (*n* = 325)
		T	23% (*n* = 99)
		CC	58% (*n* = 123)
		TC	37% (*n* = 79)
		TT	5% (*n* = 10)
	rs72558191	T	100% (*n* = 420)
		G	0%
		TT	100% (*n* = 210)
		TG	0%
		GG	0%
	rs9332131	A	100% (*n* = 422)
		AA	100% (*n* = 211)
		Del	0%
	rs9332239	C	100% (*n* = 424)
		T	0%
		CC	100% (*n* = 212)
		CT	0%
		TT	0%
	rs1057910	A	90% (*n* = 383)
		C	10% (*n* = 41)
		AA	80% (*n* = 171)
		AC	20% (*n* = 41)
		CC	0%
